# Securing Health Sensing Using Integrated Circuit Metric

**DOI:** 10.3390/s151026621

**Published:** 2015-10-20

**Authors:** Ruhma Tahir, Hasan Tahir, Klaus McDonald-Maier

**Affiliations:** Embedded and Intelligent Systems Research Laboratory, School of Computer Science and Electronic Engineering, University of Essex, Wivenhoe Park, Colchester CO4 3SQ, UK; E-Mails: htahir@essex.ac.uk (H.T.); kdm@essex.ac.uk (K.M.-M.)

**Keywords:** ICMetric, accelerometer, authentication, confidentiality, remote healthcare, secure remote password protocol

## Abstract

Convergence of technologies from several domains of computing and healthcare have aided in the creation of devices that can help health professionals in monitoring their patients remotely. An increase in networked healthcare devices has resulted in incidents related to data theft, medical identity theft and insurance fraud. In this paper, we discuss the design and implementation of a secure lightweight wearable health sensing system. The proposed system is based on an emerging security technology called Integrated Circuit Metric (ICMetric) that extracts the inherent features of a device to generate a unique device identification. In this paper, we provide details of how the physical characteristics of a health sensor can be used for the generation of hardware “fingerprints”. The obtained fingerprints are used to deliver security services like authentication, confidentiality, secure admission and symmetric key generation. The generated symmetric key is used to securely communicate the health records and data of the patient. Based on experimental results and the security analysis of the proposed scheme, it is apparent that the proposed system enables high levels of security for health monitoring in resource optimized manner.

## 1. Introduction

Healthcare devices are now increasingly being networked, thus also raising concerns about their security and safety. Attackers attempt to penetrate health monitoring devices with an intention of stealing data so that it can be used for a range of purposes ranging from medical identity theft to prescription theft. Health monitoring devices are available for a variety of purposes like medical, fitness, entertainment and lifestyle monitoring often described as “quantified self” [1]. These can be both invasive and noninvasive sensors/devices. Devices like pacemakers can be implanted into a patient’s body while smart watches and wearable sensors function externally. When these healthcare devices become part of a network like the Internet of Things, we are faced with a situation where the devices are exposed to potential attacks. There are sensors that monitor a person’s vital signs like breathing, heart rate and body temperature at regular intervals. Since these sensors monitor a person’s physiological responses, it is imperative to secure the device, data, patient and health professional in a resource efficient but comprehensive manner [2].

Traditionally, cryptographic algorithms provide security in various applications by using stored encryption/decryption keys. These algorithms have the inherent disadvantage that, if the device is compromised, the keys could be revealed to the adversaries, which would result in important data being exposed. We propose the use of Integrated Circuit Metric (ICMetric) [3] as an alternative to stored keys [4].

An advantage of the scheme presented here, is that human intervention is not required at any point during the lifecycle of its secure functioning. This is particularly important from the patient’s perspective, since this will ensure its usefulness while securely transmitting health data. Thus, the security functions of our framework are based on ICMetric data derived from the respective devices. The proposed health sensing system has the following features, which address the need for secure remote monitoring of patients:

- In this system the ICMetric technology is utilized to generate keys based on the hardware/software characteristics and specification of the device, thereby enabling device verification. This provides an effective means to address the issues related to storage of key, thereby safeguarding against the major threat of key compromise. In our work we highlight how such a device identification can be generated for a health sensor using its physical characteristics, e.g., from the Micro Electro Mechanical Systems (MEMS) accelerometer. In addition to the accelerometer credentials, we identify a range of other features that also form part of the device identification.
- In this system we propose a secure admission control process as a core component. This part of the design is aimed at recognizing each entity, which is part of the application. It recognizes each patient based on its allocated sensors, and the health professionals based on their end point device. So this part authenticates, authorizes and evaluates each entity prior to secure communication of the health data. However, at no point is the ICMetric value sent over the channel or even communicated to others.
- A challenge with the generated ICMetric is the entropy and length of the generated secret value. The proposed design generates a strong symmetric key based on the communicating parties’ device ICMetric. This generates symmetric key facilitates in secure end-to-end communications.

The remainder of this paper is organized as follows: Section 2 discusses in detail the ICMetric technology and its design principles. Section 3 provides a brief discussion on MEMS and we highlight the structure of a MEMS accelerometer as an example. This section provides details of how an ICMetric identification can be generated using MEMS accelerometers and other features specific to a health sensor. Section 4 presents the basic system design along with the design goals. The detailed design of our proposed framework for the generation of strong high entropy keys is presented in Section 5. Implementation details of the design are discussed in Section 6. Section 7 offers an in-depth analysis of the scheme from various perspectives like security, scalability and stability. This section also presents a detailed security analysis of the scheme. The paper concludes in Section 8 by summarizing the findings of this work.

### 1.1. Related Work

In [5], authors present a comprehensive survey on various aspects of Wireless Body Area Networks. In their paper, they have discussed various application scenarios, their context and specific requirements related to the technology.

Bourbakis *et al.* in [6] have proposed a mobile health platform for protecting health data during exchange with a server. This research includes various biometric authentication systems to incorporate authentication, authorization, confidentiality and integrity services in their secure symmetric health monitoring systems.

In [7], the authors proposed a scheme called Cryptographically enforced and Privacy Enhanced (CRYPE) for secure one to one communications. It uses identity based encryption for secure end to end communication and provides confidentiality and role based access control.

Yi *et al.* [8] have proposed an approach to secure communication between the sensor node and the administrator using a lightweight encryption algorithm. Furthermore, their design tries to safeguard from inside attacks by making use of the “Sharemind” system. In their proposed scheme, the authenticate-reject decision for a specific sensor is based on the outcome of three data.

Barua *et al.* [9] propose a patient centric access control scheme that guarantees integrity and confidentiality of patients health data by using digital signature and pseudo-identity techniques.

Several researchers are also looking into the design and development of key generation and key agreement schemes in BANs. The use of physiological data coupled with cryptographic schemes are two principal preferences in line with this work [10,11,12,13,14].

The development in networked health monitoring systems has led to research being carried out on resource efficient cryptographic protocols. Researchers are now decoupling the medical and security functionalities to propose resource efficient architectures executing on two separate processing elements/cores [15]. For implementing the proposed security framework; we have used AES-NI for the very purpose, to provide a lightweight scheme for remote secure health monitoring. Other works in this line include the SCAN secure processor [16], which supports secure biometric authentication and various symmetric encryption primitives.

Since the ICMetric technology is a relatively newer concept, most work has been done to prove its effectiveness and applicability. Perhaps the most extensive work on ICMetric has been done by Papoutsis. In his work [17] he proves that it is both feasible and suitable to use the hardware features of a system to generate hardware identifications. Recent studies [3] have coupled the ICMetric technology with an intelligent [18] battery powered wheelchair to provide security in health care services.

## 2. Integrated Circuit Metric (ICMetric)

Security algorithms rely on using stored secret keys. Cryptographers increase the key size to stop keys from being brute forced, in times when computation devices become cheaper and more common. Increasing the key size can deter attackers who are attempting to brute force the cryptographic keys, however this increase in key size cannot deter key theft by infiltration or simple theft. When the keys of a system are exposed, the security of the system is compromised in its entirety.

Using characteristic features specific to a device, an identification can be generated for every computation device. The device identification formally called the ICMetric is generated each time it is required and discarded thereafter thus entirely eliminating infiltration and theft based attacks. What differentiates the concept of ICMetric technology from other hardware fingerprinting techniques is the choice of features that are used for generating device identification. Conventional fingerprinting techniques rely on using features which are static, easy to capture and use. While using features that are easy to extract one is also faced with a problem that these features can be easily replicated/spoofed by attackers, thus potentially rendering the hardware fingerprint useless. Some features that can be used for generating an ICMetric are MAC addresses, CPU IDs and serial numbers. To increase the complexity of the generated ICMetric, other features are employed which are application usage specific like camera resolutions, GPS coordinates, browsing histories, common user files, system profiles *etc.* The advantage of using variable internal features is that they are difficult for an attacker to predict/spoof at runtime.

If the device fingerprint is generated by using features that are easily extractable, then the fingerprint lacks diversity and entropy. For instance even though a MAC address is a unique feature to a device, it can be extracted and hence spoofed. Some features of a device cannot be used because either they cannot be extracted or they do not adequately assist in identifying a device. The generation of the ICMetric is a two phase process, *i.e.*, calibration phase and the operation phase.

### 2.1. Calibration Phase

The calibration phase is applied once and only when the ICMetric is required. In this phase feature values are extracted from the system and normalized. In this phase the feature maps are studied for their standard deviation, confidence interval, interquartile range and skewness. A multitude of feature values are read to ensure a wide coverage of features.

### 2.2. Operation Phase

In the operation phase the feature values are combined to form an ICMetric number. Two techniques can be used for combining the individual feature values, *i.e.*, feature addition and feature concatenation [17]. In the feature addition technique the individual feature values are added to produce a shorter but highly diverse device ICMetric. The feature concatenation technique produces an ICMetric by concatenation of the individual feature values. The produced ICMetric is longer in length but lacks diversity as compared to the ICMetric generated by using feature addition technique.

### 2.3. Generating the Device ICMetric

To deter key and identification theft, the ICMetric of a device is not stored or transmitted. The ICMetric will be generated when required, therefore its generation must be comprised of lightweight mathematical functions. Generation of the ICMetric is a process requiring mathematical and statistical analysis of feature values. If a feature has a single static value that does not change, the feature does not need any form of manipulations. Whereas if the feature exhibits a range of values then the feature values need to be analyzed and studied statistically. When using the ICMetric technology the focus is on using features that have a definite range. This means that the feature values must be present in the form of clusters so that they can be placed on a normal distribution. Once this is achieved the normal distributions are analyzed, so that meaningful values can be extracted from graphical representations.

## 3. Types of Health Sensing Devices

Recent developments in various domains like telecommunication, microelectronics and health sensing has resulted in the emergence of small scale wearable devices which can detect human physiological signals with minimum manual intervention. Due to the small size of sensors it is now possible to embed them into everyday objects like clothes, wearable accessories *etc.* Health sensors can be placed into many individual categories, however it is appropriate to classify sensors based on their purpose/aspects:

- Environment—sensors designed to detect temperature, humidity, pressure, light, noise *etc.*
- Device—some sensors are designed to sense physiological data directly from the human body, while other sensors use images like facial features, eye movement to measure data.
- User—sensors designed to detect user specific data like location, acceleration, heart rates, EMI, body temperature, *etc*.
- Interaction—sensors designed to interact with multiple device through diverse mediums like RFID, Bluetooth, Ethernet *etc.* Pervasive sensors allow multiple sensors to interact with each other.

Modern devices are fundamentally a collection of many individual sensors that operate in harmony. This collection of interoperating sensors allows devices to provide extended range of services.

### 3.1. MEMS Accelerometer

MEMS is a technology that was first studied [19] in 1986 and it is now utilized in a wide range of devices [20]. MEMS technology works at micro scale to create efficient electrical systems that are based on mechanical elements. If two sensors are given the same stimuli then we can say with a high level of certainty that both sensors will provide different results [21]. This is precisely why sensor based technologies need calibrations and adjustments. This variance is due to how MEMS are manufactured. Minor imperfections in the fabrication process can have significant impact on how the sensors behave. The MEMS accelerometer is highly sensitive and imperfection prone sensor which is adversely effected by the fabrication process. Slight stresses introduce a permanent offset which varies from sensor to sensor and cannot be predicted. The use of this offset is sufficient for the establishment of a device authentication. Recent research [22,23,24,25,26] in the field of sensor based device identification proves that it is both possible and feasible to generate device identifications by using sensory data. Practically it is not always easy/feasible to extract a device imperfection. For instance to measure the offset of gyroscopes, a special device is required that can rotate the target device at a constant angular velocity. Construction of such a device is a complex task in the lab and the same behavior cannot be replicated while the sensor is put into practical use. Given in Table 1 are various sensors and their bias which can be used to form an identification.

**Table 1:** Sensors and their associated bias.

Sensor	Bias
Accelerometer	Linear acceleration bias
Gyroscope	Linear gyroscopic bias
Touchscreen	Screen misalignment
Camera	Camera noise pattern
GPS	Clock skew

### 3.2. Accelerometer Offset Measurement and Device Feature Establishment

**Figure 1:**
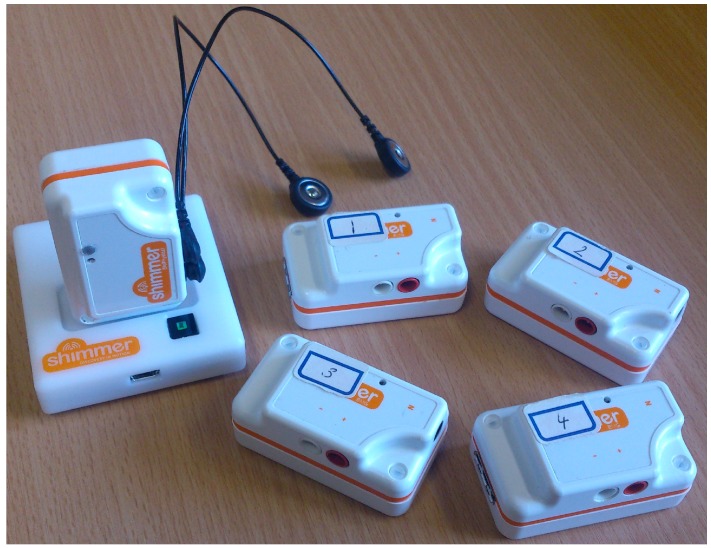
A collection of five Shimmer sensors used to determine the accelerometer bias.

To determine the offset of the accelerometer the sensing device must be placed on a stable surface which is free from vibrations and erratic movements. Using the Shimmer [26] as an experimental platform we placed the sensors on such a surface and obtained 1552 individual readings from the accelerometer sensor. The Shimmer sensor is embedded with a Freescale MMA 7361 [27] that has two selectable sensitivities, *i.e.*, ±1.5 g and ±6 g. The output CSV file is composed of a number of values that are read at regular intervals. The data provided includes a raw timestamp, calibrated timestamp (microseconds), raw accelerometer values, calibrated accelerometer reading (m/s^2^). Figure 1 shows the experimental setup composed of five individual sensors that transmit readings via Bluetooth. One sensor is plugged into the docking base station.

As highlighted above, the accelerometer is becoming a standard sensor in many computation devices. Hence, the proposed scheme for the extraction of an accelerometer offset can be used in any computation device that possesses an accelerometer sensor.

A candidate feature for the generation of an ICMetric must possess the property that its data can be normalized. This is a necessary requirement because if the data cannot be normalized, then the data cannot possess a deterministic range. Analysis of the normal distributions comprises of determining the confidence interval, standard deviation, interquartile range, variance, mean value and the skewness of the normal distribution. The confidence interval gives the guarantee that the mean of an interval is contained within the defined limits of the normal distribution. The confidence interval is an important characteristic, because if the accelerometer exhibits erratic behaviour then the mean of an individual axes will also be displaced resulting in a displaced confidence interval. To generate the device ICMetric, we have used a 95% confidence interval in order to obtain a high level of certainty. If $\bar{X}$ is the mean of the axis, n is the number of individual values in the distribution and σ is the standard deviation then the confidence interval CI is given by:

(1) $$95\%\;CI=\bar{X}\pm 1.96\frac{\sigma }{\sqrt{n}}$$

The CI is composed of a numeric multiplier of 1.96. This multiplier gives a two sided confidence interval of 95%.

Another statistical indicator which is used in the analysis of the normal distribution is the probability mass function. The probability mass function is a function f(x), which defines the probability that the random variable takes a particular value in a fixed range thus,

(2) $$f\left(x\right)=\frac{1}{\sigma \sqrt{2\pi }}e^{\frac{-{\left(x-\mu \right)}^{2}}{2\sigma^{2}}}$$

where $\sigma^{2}$ is the variance.

**Figure 2:**
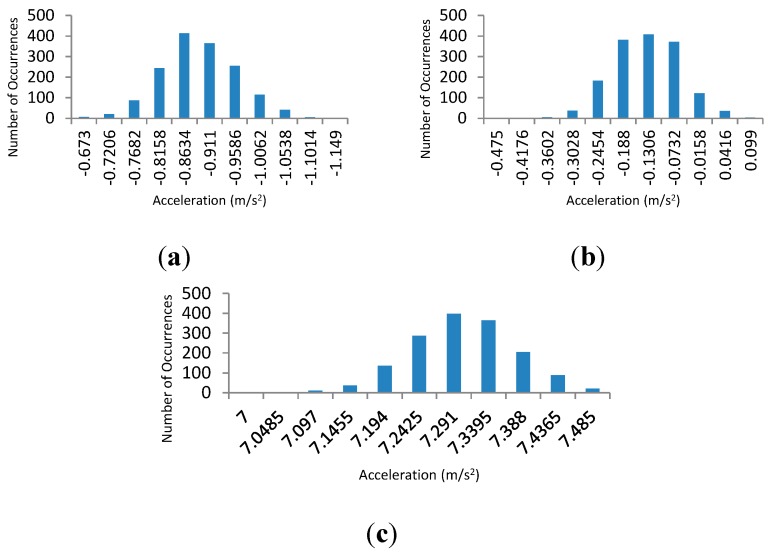
Calibrated accelerometer normalization graph for the (**a**) *x*-axis; (**b**) *y*-axis; and (**c**) *z*-axis.

The normal distribution of the readings obtained from the *x*, *y* and *z* axis show that the accelerometer readings can be normalized and used for the establishment of an ICMetric number. Statistical and mathematical analysis of the normal distributions shown in Figure 2 proves that distinct values accurately reflect the accelerometers responses along the three axes. Table 2 provides the analysis of the normal distribution for two different devices. The values show that the bias can differ dramatically, which makes it a strong metric for establishing the device ICMetric.

**Table 2:** Statistical analysis of the normal distribution for two different devices A and B.

		Device A	Device B
*x*-axis	Confidence Interval	(−0.919,−0.912)	(−0.281,−0.298)
	Standard Deviation	0.071	0.0693
	Inter Quartile Range	−0.285	−0.473
	Mean	−0.916	−0.292
	Skewness	−1.190	0.0229
*y*-axis	Confidence Interval	(−0.160,−0.167)	(−9.248,−9.271)
	Standard Deviation	0.074	0.0678
	Inter Quartile Range	0.344	0.411
	Mean	−0.163	−9.266
	Skewness	0.072	0.0685
*z*-axis	Confidence Interval	(7.284,7.276)	(−0.598,−0.621)
	Standard Deviation	0.075	0.053
	Inter Quartile Range	0.291	0.0728
	Mean	7.280	−0.615
	Skewness	−31.341	−1.371
	**Resulting ICMetric**	**−13.286**	**−31.5687**

It is this resulting statistical data which is used to form the final device ICMetric. The values are combined to generate a final ICMetric using either the feature combination or the feature addition technique discussed in Section 2.2.

**Figure 3:**
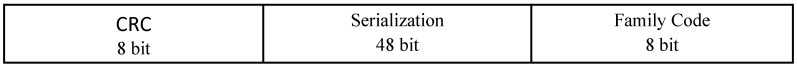
The DS-2411 serialization structure.

**Figure 4:**
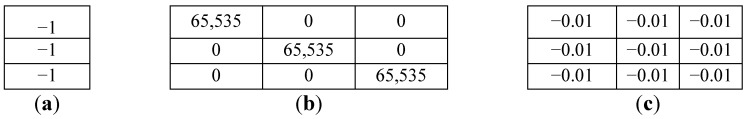
The calibration matrices with sample values. (**a**) Offset vector; (**b**) Sensitivity Matrix; (**c**) Alignment Matrix.

The ICMetric cannot be generated by just using the bias in an accelerometer. More features can be incorporated so that the generated identifier is truly diverse and ensures that the ICMetric cannot be easily predicted/spoofed. A range of features were identified, which can assist in the formation of the device ICMetric. Although these features have been studied in the Shimmer sensor, the same features can be found in other modern body sensors. Therefore the proposed features are not limited to the Shimmer sensor only. Given below is a range of features that are used for generating the ICMetric:

- Sensor Bluetooth MAC address—a 48 bit MAC address that uniquely identifies each sensor.
- Dongle Bluetooth MAC address—a 48 bit MAC address that uniquely identifies the dongle to which the sensor is connecting.
- Bluetooth radio identification—a 16 bit hexadecimal number that is associated with a device. This identification can be modified which makes it a strong ICMetric candidate.
- Factory lasered 64 bit registration—Every sensor is equipped with a DS-2411 chip [28] which provides the device with a unique identification. This is a single line chip which is factory coded with a serial that can be read for the purpose of identification. The serial number is composed of a CRC, serialization and a family code. The serialization structure is given in the Figure 3:

- Calibration Matrices—it is the role of every sensor to generate readings that are accurate. The calibration is defined by three individual matrices namely the offset vector, sensitivity matrix and alignment matrix. Figure 4 shows a sample set of calibration matrices which can be used to identify a sensor. Here it must be pointed out that the smallest change in the calibration matrices causes a chain of events which results in the wrong ICMetric being generated. When an attacker attempts to guess the calibration matrices, the resulting accelerometer readings will not reflect what was expected from the sensor. This will ultimately result in the wrong ICMetric being generated. The readings are generated following a specific algorithm [29] targeting accelerometer readings and calibrations.

The experiments using the Shimmer health sensor show that the accelerometer bias is unique and repeatable for each sensor. The *x*, *y* and *z* axis readings of a single sensor show no correlation between each other. A comparative study of our sensors shows that sensors display no particular pattern and that the bias cannot be predicted. The resulting device ICMetric is a singular device identification because the bias is an attribute of the embedded accelerometer [30]. Combining the accelerometer bias metrics with other inherent device features ensures that the ICMetric is in fact a unique device identification.

## 4. Framework Assumptions

**Figure 5:**
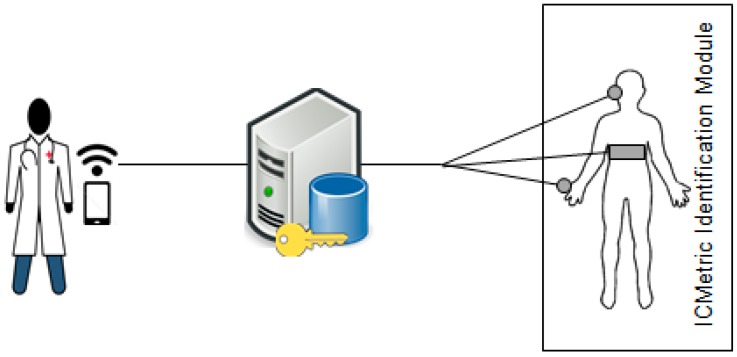
System model for an ICMetric based health monitoring system.

Attacks [31,32] on a healthcare system can target the client, KGC or network. Healthcare applications address several security and privacy challenges to secure the healthcare data. Eavesdropping is a major threat to patient privacy; the adversary can easily read unencrypted messages being transmitted over the network. Physiological data can be safeguarded from these attacks by employing stronger security measures in the system that provides privacy to the sensed patient data. The security framework proposed here seeks to improve the security of health monitoring applications by providing secure admission control, authentication, confidentiality and integrity of the health data in combination with the ICMetric technology. The proposed scheme enables networked environments and all entities/devices forming part of the network trust in a Key Generation Center (KGC). The KGC is responsible for controlling individual entities in the network. The KGC enables entities that have never had contact before to interact securely and confidentially. Figure 5 shows the general system model connecting the patient, KGC and the health professional. The KGC is operated by the healthcare provider and is responsible for assigning all the network specific configurations to successfully join a network. All entities have already decided to trust the KGC, either without authentication or on the basis of KGC authentication via SSL.

## 5. The Security Framework

The proposed security framework details a secure health sensing system which combines wearable health monitoring devices with the ICMetric technology to guarantee a system which can deter common attacks on healthcare systems. The following section details the steps involved in the secure functioning of the health monitoring application based on ICMetric:

### 5.1. Secure Admission Control

The requirement and importance of secure admission control is obvious, as key management and secure communication schemes are effective only after the devices join the network in a secure admission process. The admission control scheme is used only once, when the entity registers for the first time. The registration of an entity is a separate process that requires both manual and electronic data collections. The entity registration consists of the following unique activities:

- When an entity wishes to register with the KGC, its necessary credentials are forwarded to a KGC where the registration is digitized.
- Once the entity is registered, the KGC is updated with a unique identification and a 128 bit random per-entity value (so called “salt”).
- A copy of the identification and salt is also provided to the entity for storage.

### 5.2. Symmetric Cryptographic Module

This section provides the architectural details of symmetric key cryptographic module in the protocol design. The symmetric cryptographic module provides security functionalities that facilitate secure transmission of data between the KGC and the entity, which might be a sensor or a device. The proposed scheme is an attempt to improve the security of ICMetric by generating a symmetric session key between the sensor and the KGC, which is resilient against pre-computed attacks. The generated symmetric key is then used for securely communicating the data between the authenticated parties.

The process of symmetric key generation and authentication is carried out between the entity and the KGC to establish symmetric key for secure data communication between them. The following section details the steps involved in this process:

#### 5.2.1. Strong Symmetric Key Generation

As mentioned above in Section 5.1, the KGC stores certain entries for each registered entity (sensor, device, *etc*.) in the form of a tuple. The entity ID is the identifying attribute for an entity assigned by the KGC at the time of registration. The salt s is generated by the KGC as a random number 128 bit number at the time of registration. The KGC already has a verifier v received and stored for every entity, which it uses now during the process of symmetric key generation and authentication. The entity generated its verifier v at registration time and sent it to KGC as:

(3) $$v\;=\;g^{x_{A}}\;mod\;N$$

where g is a generator of the multiplicative group and n is a safe prime as described in Table 3.

**Table 3:** Mathematical notations.

Symbol	Meaning
||	The concatenation operator
N	A large prime number. All computations are performed modulo *n*
g	A primitive root modulo n (often called a generator)
$salt_{A,}salt_{B}$	A random string used as the entity’s and KGC’s salt
v	The verifier of the entity
$IC_{A},IC_{B}$	ICMetric of entity and KGC respectively
$ID_{A,}ID_{B\;}$	ID of entity and KGC respectively
$x_{A},x_{b}$	ICMetric number of entity and KGC respectively
a, b	Ephemeral private keys, generated randomly and not publicly revealed
h( )	One-way hash function e.g., SHA-256
u	Scrambling parameter-A and B concatenated and hashed
$K_{A},\;K_{S}$	Session keys

The design of the ICMetric symmetric key generation and authentication scheme is based on the Secure Remote Password Protocol [33,34]. SRP is particularly suitable for this application, since its properties and design works very well with the constraints of ICMetric technology of not transmitting the ICMetric number. SRP doesn’t require the exchange of ICMetric information between parties for the purpose of authentication and key generation.

To formally start the ICMetric based symmetric key generation process each registered sensor that wishes to communicate generates its own ICMetric:

(4) $$x_{A}\;=\;IC_{A}$$

The authentication process for the purpose of sending data readings to the KGC is initiated by the entity, when it sends an initiation request containing its identification to the KGC asking for the assigned salt value. On contacting the KGC, each sensor receives the salt stored on the KGC under its sensor IDA. Now the sensor generates number a:

(5) $$a\;=h\;\left(x_{A}\left|\left|\;salt_{A}\;\right|\right|\;ID_{A}\right)$$

and uses it to calculate A and sends the result to the KGC:

(6) $$A\;=\;g^{a}\;mod\;N$$

The KGC does a similar operation to calculate b:

(7) $$b\;=h\;\left(x_{B}\left|\left|\;salt_{B}\;\right|\right|\;ID_{B}\right)$$

thereby calculating B and also adds the public verifier to it and finally sending B to the sensor:

(8) $$B\;=\;\left(kv\;+\;g^{b}\right)\;mod\;N$$

where k=h(N||g).

Both sides compute a random scrambling parameter u=h (A || B) based on the exchanged A and B. So both sides can now construct the shared session key. The sensor constructs it as follows:

(9) $$\begin{array}{c} S_{B}={\left(B\;–\;k\cdot v\right)}^{a+ux}\;mod\;N\;\\ ={\left(kv+g^{b}-k\cdot v\right)}^{a+ux}\;mod\;N\;\\ ={\left(g^{b}\right)}^{a+ux}\\ K_{A}=h\;\left(S_{A}\right) \end{array}$$

The KGC constructs it as follows:

(10) $$\begin{array}{c} S_{B}={\left(A\cdot v^{u}\right)}^{b}={\left(g^{a+xu}\right)}^{b}\\ K_{B}=h\;\left(S_{B}\right) \end{array}$$

Both sides now possess the same secure session key K based on the respective formulae.

#### 5.2.2. Symmetric Key Authentication

To complete the authentication, now the sensor needs to prove to KGC that its key is identical. In order to do so, the sensor constructs the message $M_{1}$ and sends it to the KGC:

(11) $$M_{1}=h\;\left(\;N\;\left|\left|\;g\;\right|\right|\;ID_{A}\;\left|\left|salt_{A}\;\left|\left|\;A\;\right|\right|\;B\;\right|\right|\;K_{A}\;\right)$$

**Figure 6:**
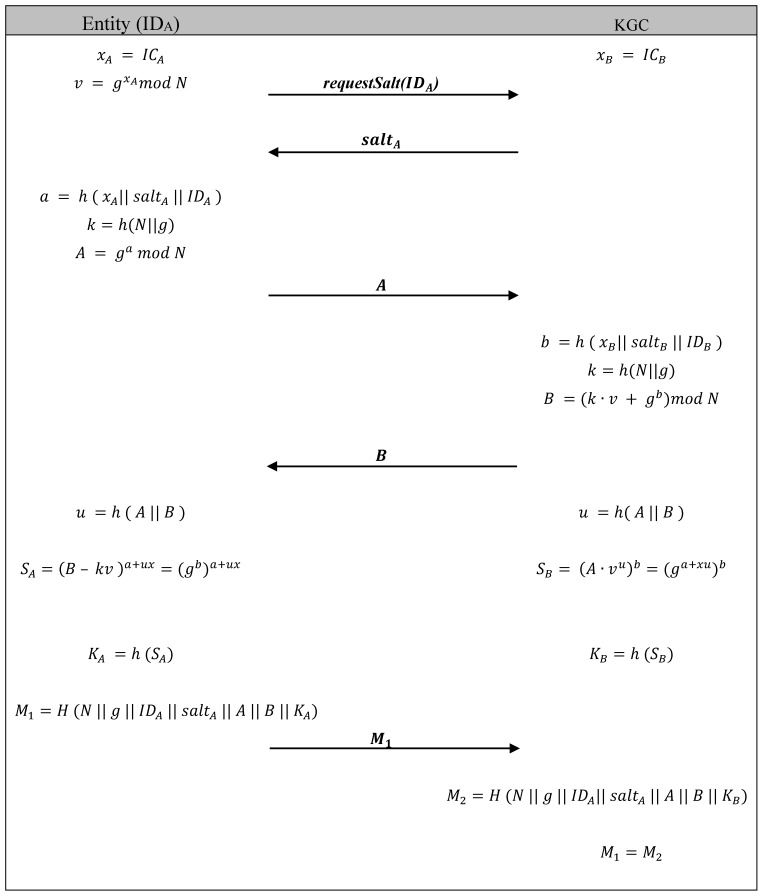
ICMetric based symmetric cryptographic module.

The KGC will calculate $M_{2}$ using its own $K_{S}$ and compare it against the message received from the sensor. If both keys do not match, the authentication fails resulting in refusal of communication request. If the sensor request is authenticated, the data can safely be relayed to the KGC. The summarized protocol is given in Figure 6. After an entity is authenticated and joins the network, its session key is kept in a secure cache and is valid for a set time period.

#### 5.2.3. Symmetric Key Encryption/Decryption

Once the session key is in place, secure communications can be carried out using any well-known symmetric key encryption algorithms such as AES or 3DES. Here we propose using AES to securely send health data between the entities and KGC. So in this step of the design we integrate all the individual ICMetric based components with the symmetric encryption/decryption scheme to achieve data confidentiality and integrity between the parties. This module performs encryption/decryption of the health data between the sensors and the KGC.

## 6. Implementation

A working prototype of the health sensing system was implemented using C on Linux. The scheme has been implemented on a first generation Intel Core i3 3.2 GHz processor with 6 GB RAM. The authentication and key generation module of the design has been implemented using the openssl cryptographic library. For the implementation of symmetric encryption/decryption we have used the CyaSSL library. Although there is support for AES in OpenSSL, but we use the CyaSSL library for implementing the encryption/decryption counterpart of the design. The CyaSSL [35] library is a lightweight SSL library that specifically targets resource constrained devices. CyaSSL is 20 times smaller than the standard OpenSSL. CyaSSL also takes advantage of Intel AES-NI support [36]. This helps boost the performance of AES, since the instructions execute directly on the chip instead of executing the algorithm from software, thereby making it run 5–10 times faster. We intend to deploy this scheme on an embedded system. The design and implementation choices are greatly influenced by the fact that embedded platforms possess limited resources. Detailed results in the upcoming section also show that the scheme can achieve high levels of security without resource demand when deploying on a real sensor test bed.

The authentication and key generation module of the system implementation has five variants namely 160, 224, 256, 384, 512 bits; with a 128-bit device ICMetric as input. To offer flexibility the encryption/decryption module of the scheme has been implemented to facilitate two AES variants namely AES-128 and AES-256.

## 7. Results and Discussion

In this section, the efficiency of the design is evaluated by measuring the RAM consumption and execution time of the implemented scheme. For the purpose of evaluating the execution time of the system the programs runtime itself is utilised, whereas for the purpose of evaluating the memory consumed by the prototype Valgrind tool was employed.

Valgrind [37] is a code profiling and memory debugging tool for Linux that employs Massif for memory profiling. Massif [38] analyses the implementation and measures how much memory the program uses during its lifetime and classifies this as follows:

- Useful heap—heap used by the program over time.
- Extra heap—heap blocks allocated for administration data over the program’s lifetime.
- Total heap—total of the useful heap and extra heap allocated for the program over its lifetime.
- Stack—stacks used by the program over time.

### 7.1. Performance Evaluation of ICMetric Key Generation and Authentication Module

#### 7.1.1. Execution Time

The time requirements for authentication and the key generation counterpart, which are responsible for the authentication and the key generation of an ICMetric based symmetric key, are evaluated. A 128 bit ICMetric is utilised as input for testing all the variants of the prototype.

**Figure 7:**
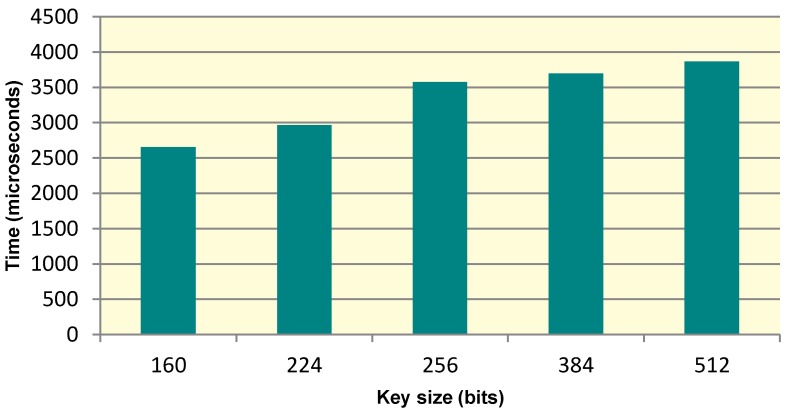
Execution times for ICMetric based symmetric key variants.

To prove the scalability of the key generation scheme, the time taken for the generation of keys of varying key sizes was evaluated. Figure 7 shows the key size *versus* time taken graph for computing key variants namely 160, 224, 256, 384, 512 bits. It is evident from the graph that an increase in the key size does not bring a drastic change to the time performance of the application. Hence, the scheme is able to provide higher levels of security without substantial execution time overheads.

#### 7.1.2. RAM Consumption

Here, the memory performance of ICMetric based symmetric key authentication and key generation counterpart has been evaluated. Once again, a 128 bit ICMetric is utilized as input for testing all the variants of the prototype. The memory performance of the ICMetric based authentication and key generation counterpart is evaluated using Valgrind. The generated graphs in Figure 9 depict memory profile of the five ICMetric symmetric key variants, by presenting the program lifecycle and the memory (bytes) consumed.

**Figure 8:**
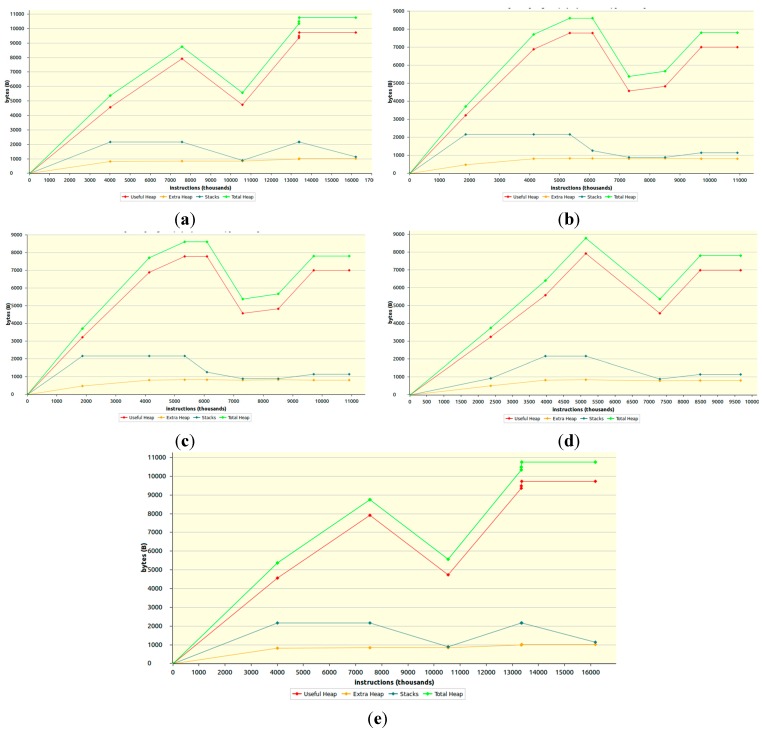
RAM consumption for ICMetric based symmetric key variants (**a**) 160-bit; (**b**) 224-bit; (**c**) 256-bit; (**d**) 384-bit; (**e**) 512-bit.

**Figure 9:**
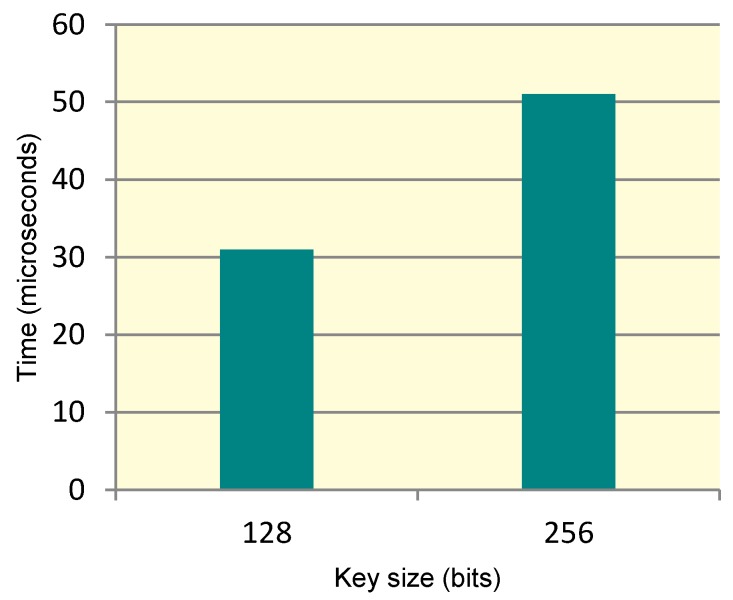
Execution times for ICMetric based AES variants.

Figure 9a shows that the maximum total memory consumed for the generation and authentication of shortest 160-bit key is around 10KB; while Figure 8e shows that the maximum total memory consumed for the generation and authentication of longest 512-bit key is around 11 KB. This proves that an increase in the key size has a very small effect on the memory consumption of the application. The graphs in Figure 8 clearly demonstrate that the health sensing scheme presented here, is able to provide high levels of security at the cost of a very limited memory footprint.

### 7.2. Performance Evaluation of ICMetric Based AES Encryption/Decryption Module

#### 7.2.1. Execution Time

In this section, the time performance of encryption/decryption module that is responsible for carrying out the encryption/decryption of a data block using the ICMetric symmetric key is evaluated. Figure 9 shows a bar graph in which the 128 and 256 bit AES key variants *vs.* the time taken. It is evident from the graph that an increase in the key size has a limited effect on the execution time.

#### 7.2.2. RAM Consumption

**Figure 10:**
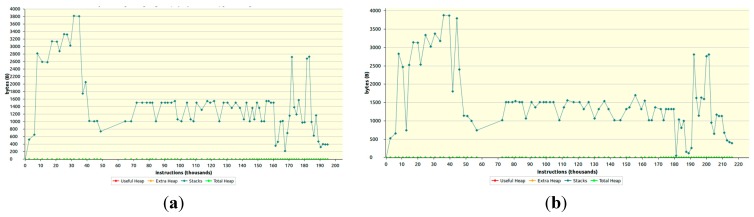
RAM consumption for ICMetric based AES encryption/decryption (**a**) 128-bit; (**b**) 256-bit.

The graphs in Figure 10a,b present memory profile for the ICMetric based 128-bit and 256-bit AES encryption/decryption, respectively.

It is apparent from the graphs in the figure that the maximum total memory consumed for the ICMetric based AES encryption/decryption using 128-bit key is around 3.8 KB and for the 256-bit key is 4 KB, which is very suitable for resource constrained devices. Therefore the proposed scheme is able to provide higher levels of security without substantial memory performance overheads.

### 7.3. Discussion

In the following section we perform a security analysis of the designed secure health sensing scheme with respect to the security goals accomplished, while also identifying the limitations of the proposed framework. Table 4 gives a summary overview of the goals accomplished/unaccomplished by individual system modules of the health sensing scheme presented in this paper.

**Table 4:** System modules and goals accomplished.

		System Goals					
		Authentication	Confidentiality	Access Control	Non Repudiation	Precomputed Attacks	Integrity
Modules	MEMS Accelerometer	√					
	ICMetric			√	√		
	Strong Key Generation					√	
	Symmetric Module	√		✓		√	√
	AES		√				√

The health sensing framework combines the security advantages of the device ICMetric and the designed symmetric key generation scheme for the generation of strong symmetric keys between the entities and the KGC. Non repudiation is an inferred security goal of the scheme, since generating the ICMetric from a range of features that are unique to a computation device prevents an entity from denying a particular action performed by their device. The original ICMetric scheme [17] has inherent weaknesses like insufficient length and entropy, owing to which it is not well suited to serve as a key for cryptographic operations. The symmetric key generation scheme in our security framework uses key derivation functions on ICMetric number coupled with a salt, to generate a strong symmetric ICMetric key. Following their use, the ICMetric and the cryptographic keys are discarded hence deterring all forms of key capturing. Each device concatenates its ICMetric with a random per-user value (so called “salt”) and stores the hash value of the result along with the salt. This makes certain kinds of brute-force attacks and dictionary attacks more difficult. The security features of the design also prevent the possibility of man in the middle attack. The scrambling parameter in the symmetric cryptographic module ensures that each entity only gets one verification attempt at impersonation, resulting in the ruling out the possibility of man-in the middle attack.

A major advantage provided by the health sensing application is that of authenticating entities without the need for human intervention. This is particularly important from the patient’s perspective, since human intervention is not always possible for authentication, therefore the authentication functionality is automatically carried out based on the sensors’ ICMetric keys. To generate the symmetric key and carry out the authentication/access control, the framework effectively conveys a zero knowledge password proof. This property is guaranteed by the symmetric cryptographic module and ensures that the entity is able to prove to the KGC about the knowledge of the ICMetric number, without actually transmitting/communicating the ICMetric to the verifying party. The data sent by each entity is encrypted using AES based on the ICMetric symmetric key, so that health data is not leaked to unauthorized parties. Integrity checking is a further functionality provided by the health sensing scheme during the secure transmission of data between the patient and the health professional. As shown in Table 4, the symmetric cryptographic module and the AES module are responsible for ensuring the integrity of computed symmetric key and communicated patient data.

## 8. Conclusions

Wearable healthcare devices have revolutionised the way in which healthcare is delivered. Wearable computing has now made it possible to deliver healthcare beyond the confines of the hospital. While there are many advantages of monitoring a person’s health remotely, a major hurdle with this healthcare model is the provision of security. In this paper an ICMetric based health sensing scheme is proposed that employs the MEMS accelerometer and other device characteristics to generate a sensor identification called the device ICMetric. This device ICMetric possesses the quality that it is generated without human intervention and only when it is required. After use, the device ICMetric and all related data are removed from the system. In the proposed scheme the functionalities of the ICMetric are combined with a symmetric key protocol for providing access control, authentication and confidentiality of health data in a resource efficient manner. By using the ICMetric technology no form of credentials are residing on a system; thus enabling to prevent major threats like key theft, man-in-the-middle attack and brute force attack related to secure communication of health data. The design decisions made in this framework have also enabled it to be particularly suitable for resource constrained environments, which require elimination of computationally expensive and resource intensive operations while providing the required security. The security analysis and implementation results of this lightweight design prove that the protocol presented in this paper is suited greatly for health monitoring applications in terms of security and resource consumption. In future, we plan to carry out practical evaluation of the proposed security framework on real sensor testbed. The issues related to availability of the proposed scheme are currently not within the scope of this work. Denial of service attacks target the availability of a system, thus future plans include also addressing this goal.

## References

[1] Vandrico Inc. (2008) Wearable Technology Database. http://vandrico.com/database.

[2] Zheng Y.L., Ding X.R., Poon C.C.Y., Lo B.P.L., Zhang H., Zhou X.-L., Yang G.Z., Zhao N., Zhang Y.-T. (2014). Unobtrusive sensing and wearable devices for health informatics. IEEE Trans. Biomed. Eng..

[3] Kovalchuk Y., Howells G., McDonald-Maier K. (2014). Overview of ICMetrics technology—Security infrastructure for autonomous and intelligent healthcare system. Intern. J. Serv. Sci. and Technol..

[4] Tahir R., Hu H., Gu D., McDonald-Maier K., Howells G. A Scheme for the Generation of Strong ICMetric Based Session Key Pairs For Secure Embedded System Applications. Proceeding of the International Conference on Advanced Information Networking and Applications.

[5] Alam M.M., Hamida E.B. (2014). Surveying wearable human assistive technology for life and safety applications: standards, challenges and opportunities. Sensors.

[6] Bourbakis N., Pantelopoulos A., Kannavara R., Nikita K. (2014). Security and Privacy in Biomedical Telemetry: Mobile Health Platform for Secure Information Exchange.

[7] Ragesh G.K., Baskaran K. CRYP: Towards Cryptographically Enforced and Privacy Enhanced WBANs. Proceeding of the First International Conference on Security of Internet of Things.

[8] Yi X., Willemson J., Nait-Abdesselam F. Privacy-Preserving Wireless Medical Sensor Network. Proceeding of the 12th IEEE International Conference on Trust, Security and Privacy in Computing and Communications.

[9] Barua M., Liang X., Lu R., Shen X. PEACE: An Efficient and Secure Patient-Centric Access Control Scheme for eHealth Care System. Proceedings of the IEEE Conference on Computer Communications Workshops.

[10] Rostami M., Juels A., Koushanfar F. Heart-to-Heart (H2H): Authentication for Implanted Medical Devices. Proceeding of the 20th ACM Conference on Computer and Communications Security.

[11] Kannavara R., Mertoguno S., Bourbakis N. (2011). SCAN secure processor and its biometric capabilities. J. Electron. Imaging.

[12] Venkatasubramanian K.K., Banerjee A., Gupta S.K.S. (2010). PSKA: Usable and secure key agreement scheme for body area networks. IEEE Trans. Inf. Technol. Biomed..

[13] Jurik D., Weaver A.C. Securing Mobile Devices with Biotelemetry. Proceeding of the 20th International Conference on Computer Communications and Networks.

[14] Venkatasubramanian K.K., Banerjee A., Gupta S.K.S. EKG—Based Key Agreement in Body Sensor Networks. Proceeding of the 2nd Workshop on Mission Critical Networks.

[15] Strydis C., Seepers R.M., Peris-Lopez P., Siskos D., Sourdis I. (2013). A System Architecture, Processor and Communication Protocol for Secure Implants. ACM Trans..

[16] Hu C., Xiuzhen C., Zhang F., Wu D., Liao X., Chen D. OPFKA: Secure and Efficient Ordered Physiological Feature Based Key Agreement for Wireless Body Area Networks. Proceedings of the 32nd IEEE International Conference on Computer Communications.

[17] Papoutsis E. (2009). Investigation of The Potential of Generating Encryption Keys For ICMetrics. Ph.D. Thesis.

[18] Kokosy A., Floquet T., Howells G., Hu H., Pepper M., Sakel M., Donze C. SYSIASS—An Intelligent Powered Wheelchair. Proceedings of the 1st International Conference on Systems and Computer Science.

[19] Defence Advanced Research Project Agency, Micro Electro-Mechanical Systems (MEMS) (1998). Annu. Rev. Fluid Mechan..

[20] Castoldi L. The MEMS Revolution (2012, September). http://www.semi.org/eu/sites/semi.org/files/docs/STM.pdf.

[21] Kuc R. (2014). Electrical Engineering in Context: Smart Devices, Robots & Communications.

[22] Feng M., Fukuda Y., Mizuta M., Ozer E. (2015). Citizen sensors for SHM: Use of accelerometer data from smartphones. Sensors.

[23] Dey S., Roy N., Xu W., Choudhury R.R., Nelakuditi S. AccelPrint: Imperfections of Accelerometers Make Smartphones Trackable. Proceedings of the 21st Annual Network and Distributed System Security Symposium NDSS.

[24] Mobile Device Identification via Sensor Fingerprinting. https://crypto.stanford.edu/gyrophone/sensor_id.pdf.

[25] Aysu A., Ghalaty N.F., Franklin Z., Yali M.P., Schaumont P. Digital Fingerprints for Low-Cost Platforms Using MEMS Sensors. Proceedings of the Workshop on Embedded Systems Security.

[26] Burns A., Greene B.R., McGrath M.J., O’Shea T.J., Kuris B., Ayer S.M., Stroiescu F., Cionca V. (2010). SHIMMER™—A wireless sensor platform for noninvasive biomedical research. IEEE Sens. J..

[27] Freescale Semiconductor (2008, April) ±1.5g, ±6g Three Axis Low-g Micromachined Accelerometer. Technical Data. http://www.freescale.com/files/sensors/doc/data_sheet/MMA7361L.pdf.

[28] Maxim Integrated DS2411 Silicon Serial Number with VCC Input (2003, May). Data Sheet. http://datasheets.maximintegrated.com/en/ds/DS2411.pdf.

[29] Ferraris F., Grimaldi U., Parvis M. (1995). Procedure for effortless in-field calibration of three-axial rate gyro and accelerometers. Sens. Mater..

[30] Tahir H., Tahir R., McDonald-Maier K. Securing MEMS Based Sensor Nodes in the Internet of Things. Proceedings of the Sixth International Conference on Emerging Security Technologies.

[31] Almulhem A. (2012). Threat modeling for electronic health record systems. J. Med. Syst..

[32] Wu T. The Secure Remote Password Protocol. Proceedings of the 1998 Internet Society Network and Distributed System Security Symposium.

[33] (2007). RFC 5054, Using the Secure Remote Password (SRP) Protocol for TLS Authentication. http://www.rfc-editor.org/info/rfc5054.

[34] CyaSSL User Manual. User Manual (2013, September). http://www.yassl.com/documentation/CyaSSL-Manual.pdf.

[35] Gueron S. (2012). Intel Advanced Encryption Standard (AES) New Instructions Set. Intel. Corp. White Pap..

[36] The Valgrind Quick Start Guide. User Manual (2014, September). http://valgrind.org/docs/manual/valgrind_manual.pdf.

[37] Wolff M. (2011, August) Massif-Visualizer Memory Profiling UI. Presentation. https://desktopsummit.org/sites/www.desktopsummit.org/files/massif-visualizer.pdf.

[38] Electronic Authentication Guideline—Recommendations of the National Institute of Standards and Technology (2006, April). http://csrc.nist.gov/publications/nistpubs/800-63/SP800–63V1_0_2.pdf.

